# Eccentric vs. Concentric Training: A Systematic Review and Meta-Analysis of Randomized Controlled Trials on Performance and Health Benefits Across Diverse Populations

**DOI:** 10.3390/sports14030119

**Published:** 2026-03-18

**Authors:** Carolina Oassé Paulafreitas Maia, Diego Ignácio Vanezuela Pérez, Rafael Pereira Azevedo Teixeira, Ciro José Brito, Esteban Aedo-Muñoz, Bianca Miarka

**Affiliations:** 1 Laboratório de Psicofisiologia e Performance em Esportes e Combates, Departamento de Lutas, Programa de Pós-Graduação em Educação Física, Universidade Federal do Rio de Janeiro, Rio de Janeiro 21941-599, Brazil; 2 Facultad de Salud y Ciencias Sociales, Universidad de las Américas, Santiago 7500975, Chile; 3 Departamento de Educação Física, Programa de Pós-Graduação em Educação Física, Universidade Federal de Juiz de Fora, Juiz de Fora 36036-900, Brazil; cirojbrito@gmail.com; 4 Departamento de Educación Física, Deportes y Recreación, Universidad Metropolitana de Ciencias de la Educación, Ñuñoa 7760197, Chile; esteban.aedo@gmail.com

**Keywords:** athletic training, rehabilitation, muscle strength, hypertrophy, cardiovascular health

## Abstract

Eccentric (ECC) and concentric (CON) muscle training produce distinct physiological responses, with potential implications for musculoskeletal, metabolic, and cardiovascular health. Therefore, our objective is to synthesize evidence from randomized controlled trials comparing the effects of ECC and CON training on strength, hypertrophy, metabolic function, and cardiovascular health across diverse adult populations. A systematic review and meta-analysis were conducted in accordance with PRISMA guidelines and registered in PROSPERO (ID: CRD42024627600). The review included eight randomized controlled trials, pooling data from a total of 441 participants. For strength-related outcomes, six studies (n = 322) were included; for hypertrophy, four studies (n = 210); and for cardiovascular measures, three studies (n = 154). Studies were assessed using the TESTEX scale. Standardized mean differences and random-effects models were applied (*p* ≤ 0.05). Results indicated that ECC training consistently produced moderate to large improvements in muscle strength (pooled ES = 0.95; I^2^ = 78.6%) and hypertrophy (pooled ES = 0.60; I^2^ = 62.3%), particularly in populations with chronic obstructive pulmonary disease (COPD) and older adults. The rate of force development (RFD) showed large effect sizes for ECC (RFD50: ES = 0.97; RFD100: ES = 0.95) but minimal change for CON (RFD50: ES = 0.04; RFD100: ES = 0.10). Both ECC and CON showed minimal effects on cardiovascular outcomes (heart rate and blood pressure: pooled ES range = −0.16 to 0.00; I^2^ = 41.8%) and limited tendon remodeling (ES = −0.18). In conclusion, ECC exercise demonstrates superior benefits for improving muscular strength, hypertrophy, and power across varied populations, particularly those with clinical conditions such as COPD. Its impact on cardiovascular health and tendon properties, however, appears limited. These findings support the integration of ECC modalities into targeted rehabilitation and performance programs.

## 1. Introduction

The practice of physical exercise plays a critical role in maintaining performance and health, serving as a cornerstone for preventing and treating chronic cardiorespiratory and metabolic diseases, while mitigating age-related muscle loss [1,2]. According to the World Health Organization, cardiovascular diseases (CVDs) are on the rise, chronic obstructive pulmonary disease (COPD) ranks as the third leading cause of death globally, and osteoarthritis (OA) affects over 10 million people, making it one of the primary causes of physical disability in Western societies. With an aging population, the incidence of CVD, COPD, and OA is expected to increase [3,4,5].

Aging is associated with a decline in physical fitness levels, leading to reduced muscle mass, particularly in the quadriceps. This decline in muscle mass is linked to decreased lower-limb strength, resulting in impaired balance, stability, and mobility, as well as changes in tendon mechanical properties [5,6]. Patients with COPD experience a more pronounced reduction in lower-limb muscle strength compared to healthy individuals and frequently develop cardiovascular and metabolic complications, such as hypertension and insulin resistance [4]. Furthermore, aging is closely associated with increased arterial stiffness, a significant risk factor for cardiovascular events [3]. Research has demonstrated that physical training is a key public health intervention for reducing and managing chronic diseases. It improves metabolic and cardiorespiratory health, prevents muscle atrophy, enhances functional capacity, and promotes reintegration into daily activities, thereby fostering autonomy [1,2,3].

Adherence to resistance training has been linked to a 21% reduction in mortality rates [7]. In recent years, research has increasingly focused on the benefits of eccentric (ECC) training compared to concentric (CON) training, emphasizing its advantages across various populations. These include healthy individuals, athletes, and those with physical limitations, such as those undergoing cardiac rehabilitation or living with COPD or OA [2,5,7]. It is well-established that muscular strength and cardiovascular endurance are significantly influenced by the type of muscle contraction performed during exercise [2,8,9,10,11,12,13,14,15,16,17,18,19].

ECC and CON muscle actions represent two fundamental modalities of resistance training with distinct physiological and functional properties. ECC exercise, characterized by muscle tension during active lengthening, is considered particularly effective due to its lower metabolic cost, allowing for high-intensity exercises with reduced cardiovascular strain and perceived effort [9,15,16,17] These physiological differences help explain why ECC is often more tolerable for individuals with limited exercise capacity and may promote greater long-term adherence to exercise programs compared to traditional rehabilitation methods that focus predominantly on CON actions. From a mechanical perspective, ECC actions display a higher force-to-tension ratio, selective regional hypertrophy, and architectural remodeling of muscles, including adaptations in sarcomere and fascicle length [20]. Unlike CON contractions, ECC actions paradoxically generate greater force as execution velocity increases and rely on unique morphological structures such as titin, fascia, aponeuroses, and tendons [20]. Functionally, the musculotendinous system acts as both a shock absorber and an elastic spring, dissipating impact forces or storing elastic energy during stretch–shortening cycles (SSC). This makes ECC particularly relevant in sports and daily tasks involving landings, decelerations, or rapid changes of direction, contributing not only to performance but also to the protection of passive anatomical structures [20].

**Figure 1:**
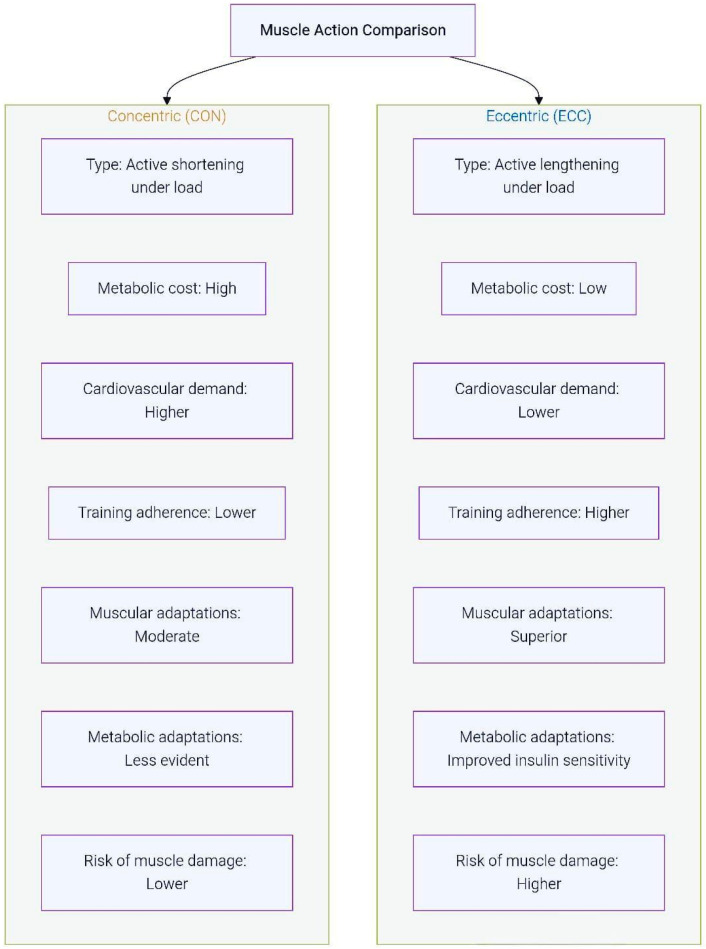
ECC vs. CON Training: Key Physiological and Functional Differences.

In terms of adaptations, ECC training has been shown to enhance maximal strength, rate of force development, hypertrophy, and muscular power, while also promoting favorable metabolic effects such as improved insulin sensitivity and glycemic control [1,7,17,20]. These characteristics support its application in sports performance, rehabilitation, and clinical populations, including older adults and patients with chronic conditions such as sarcopenia, cachexia, and type 2 diabetes [1]. At the same time, ECC exercise is associated with greater muscle damage and delayed-onset muscle soreness (DOMS) compared to CON and isometric modalities, emphasizing the need for careful prescription of intensity, frequency, and progression [7]. Taken together, ECC and CON exercises should be viewed as complementary modalities: while CON work is metabolically demanding and essential for propulsion and endurance tasks, ECC provides unique mechanical and adaptive benefits that make it indispensable for strength development, rehabilitation, and injury prevention (Figure 1).

While ECC has proven effective in various clinical and athletic settings, the impact of training variables such as intensity, frequency, and duration remains unclear. Inconsistencies observed in outcomes for individuals with chronic diseases and older adults suggest a complex paradigm: ECC may not yield universally positive effects across all populations and protocols.

Studies assessing ECC often employ a combination of measures, including muscular strength, body composition, functional performance, and metabolic parameters. Muscle strength is frequently evaluated using isometric and dynamic tests, such as maximum voluntary contraction (MVC) or maximal ECC contraction force measurements. For instance, Yoshida et al. [18] assessed elbow flexor strength in participants undergoing ECC training using an isokinetic dynamometer. Body composition assessments often involve methods such as bioelectrical impedance analysis or computed tomography (CT) to evaluate changes in lean mass and body fat following training. Functional quality, particularly in populations with comorbidities, is commonly assessed using tests like the 6-Minute Walk Test (6MWT) or the Timed Up and Go (TUG) test, which evaluate participants’ mobility and endurance before and after ECC interventions [2,4].

In addition to muscular and functional outcomes, studies have examined ECC’s effects on cardiovascular and metabolic parameters, such as blood pressure, heart rate, and insulin sensitivity. For example, Faivre-Rampant et al. [9] evaluated cardiovascular responses to ECC and CON in COPD patients using blood pressure monitors and heart rate trackers to measure physical effort and recovery during exercise. Similarly, Touron et al. [1] investigated the impact of ECC on endothelial function and glycemia, aiming to understand its metabolic benefits for individuals with obesity and type 2 diabetes. These assessments are essential for determining ECC’s efficacy in improving muscle strength and its broader implications for overall health and the prevention of chronic disease complications [9,10,11,12,13,14,15,16]. The diversity of evaluation methods underscores the complexity of ECC responses and the need for comprehensive analysis to fully understand its therapeutic potential [21,22].

We hypothesize that ECC exercise is more effective than CON exercise in promoting muscle strength and hypertrophy, particularly in populations with comorbidities, such as COPD patients and older adults. Therefore, this study aims to validate the superiority of ECC compared to CON across various populations and its implications for musculoskeletal, cardiorespiratory, and metabolic health. By exploring the necessary training intensities and conditions, this comparison seeks to provide a deeper understanding of the impact of each exercise type on muscle function, cardiovascular health, and metabolism. It also aims to guide exercise prescription by elucidating the factors that optimize or limit the benefits of ECC.

## 2. Materials and Methods

### 2.1. Data Collection and Analysis

This systematic review and meta-analysis adhered to a rapid review methodology, designed to provide timely and evidence-based insights into the effects of ECC versus CON training. The study protocol was registered in PROSPERO (ID: CRD42024627600), an international database of prospective health-related study protocols, including systematic, rapid, and comprehensive reviews. Ethical approval was deemed unnecessary due to the lack of human participation or patient data use. No deviations from the registered protocol were made, and all planned methods and analyses were conducted as specified in the PROSPERO registration.

The review followed the PRISMA (Preferred Reporting Items for Systematic Reviews and Meta-Analyses) guidelines. PRISMA ensures transparency and comprehensiveness in reviews, incorporating key elements such as detailed search strategies, study selection criteria, and data synthesis. This approach was adapted to suit the objectives of this rapid review while maintaining high methodological standards.

The study applied the PICO framework to structure the clinical question and review process (Population: Adults aged ≥19 years; Intervention: ECC training; Comparator: CON training; Outcome: Effects on muscle strength, hypertrophy, body composition, and metabolic and functional parameters).

### 2.2. Search Strategy

A comprehensive search was conducted in PubMed, Scopus, and SPORTDiscus™, targeting studies that compared the effects of ECC and CON training. The following search terms and combinations were used:

PubMed: (“eccentric exercise” OR “eccentric contraction” OR “eccentric training”) AND (“muscle damage” OR “delayed onset muscle soreness” OR “DOMS” OR “muscle recovery” OR “muscle hypertrophy” OR “muscle adaptation” OR “inflammation”) AND (“body composition” OR “caloric expenditure” OR “energy expenditure” OR “strength improvement” OR “muscle strength”) AND (“athletes” OR “sedentary individuals” OR “elderly” OR “adults” OR “women” OR “men” OR “rehabilitation”).

Scopus: (“eccentric exercise”) AND (“muscle damage” OR “delayed onset muscle soreness” OR “DOMS”) AND (“body composition” OR “energy expenditure” OR “muscle strength”) AND (“sedentary individuals” OR “elderly”).

SPORTDiscus™: (“eccentric exercise”) AND (“muscle damage” OR “DOMS”) AND (“body composition”) AND (“sedentary individuals” OR “elderly”) NOT (“review” OR “meta-analysis” OR “chapter”).

This search yielded eight original studies directly evaluating ECC exercise as the primary experimental variable. Only randomized controlled trials (RCTs) were included. Quasi-experimental, observational, case series, and review articles were excluded. The literature search covered studies published from January 1990 to 9 October 2024. Studies were included if published in English; articles in other languages were excluded due to the lack of translation resources. The inclusion and exclusion criteria were defined based on the PICO framework. Inclusion criteria: Adults aged 19 years or older, and studies comparing ECC and CON training effects. While exclusion criteria were Articles not in English; Review articles, abstracts, or non-primary research, and; Studies with confounding variables, such as aerobic exercise programs, combined training interventions, dietary protocols, or pharmacological treatments. Non-English records were excluded via filters, which may have introduced language bias by omitting relevant evidence published in other languages. Present research restricted inclusion to studies published in English due to limited translation resources. This approach ensured consistency in data screening and extraction and reduced potential heterogeneity arising from different reporting styles, outcome definitions, or translation errors.

### 2.3. Study Screening

The initial search results were screened based on titles and abstracts. Potentially relevant studies underwent full-text reviews to confirm eligibility. The screening process was conducted using the Rayyan web tool, which facilitates systematic review workflows by organizing and filtering studies.

#### 2.3.1. Data Extraction and Management

Data from the included studies were systematically extracted using a standardized spreadsheet. Key information recorded included: Participant characteristics (age, health status, training background); Intervention details (ECC or CON protocols, intensity, duration, frequency); Study methodologies, and: Outcomes (muscle strength, hypertrophy, body composition, functional capacity, metabolic parameters). When data were missing or incomplete, corresponding authors were contacted by email (up to two attempts) to request additional information. If data could not be obtained, results were either excluded from meta-analysis or imputed, where appropriate, using standard methods (e.g., calculating missing standard deviations from confidence intervals or *p*-values).

#### 2.3.2. Primary Results

**Figure 2:**
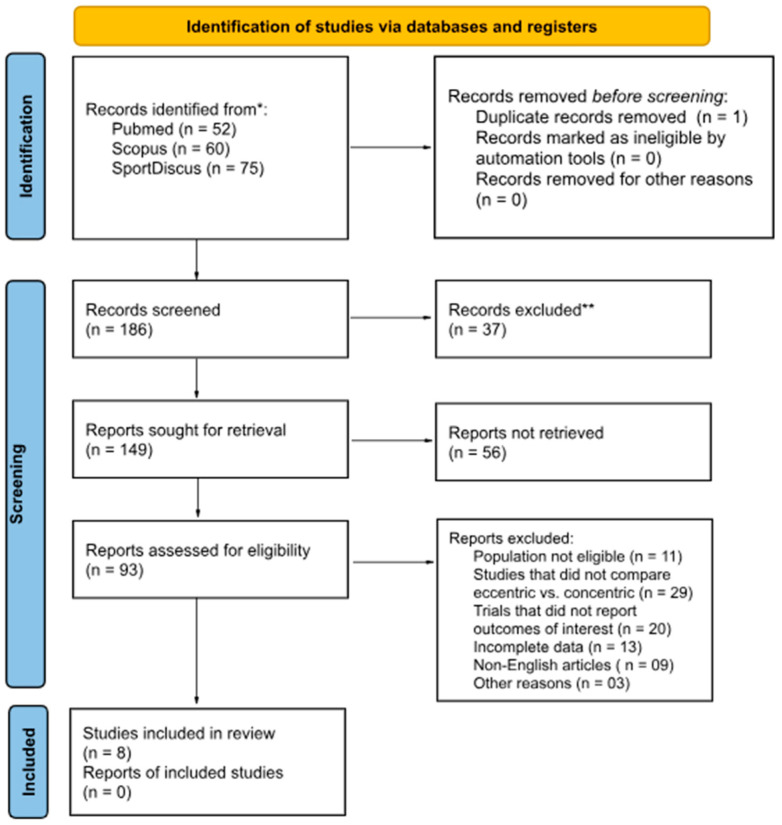
PRISMA 2020 flow diagram showing study selection. At the eligibility stage, 93 full-text articles were assessed, of which 85 were excluded. The most common reasons for exclusion were: ineligible population, absence of eccentric versus concentric comparison, trials that did not report outcomes of interest, incomplete data, non-English language, and other study design issues. **Note:** * Studies identified in one or more of the platforms selected for the study; ** Records excluded correspond to studies removed during the title and abstract screening phase because they clearly did not meet the predefined inclusion criteria. These exclusions were made prior to full-text retrieval and therefore did not require detailed justification.

Two reviewers (Author 1 and Author 2) independently screened all titles and abstracts for eligibility. They were blinded to each other’s decisions during the initial screening stage. Full-text screening was then conducted independently by the same two reviewers, with discrepancies resolved by discussion or consultation with a third reviewer (Author 5). Figure 2 shows the PRISMA 2020 flow diagram. PRISMA checklist in Table S1.

### 2.4. Qualitative Analysis

Two reviewers independently assessed methodological quality using the TESTEX scale. Disagreements were resolved through discussion or, if necessary, arbitration by a third reviewer. To complement the TESTEX evaluation and align with CONSORT recommendations for RCTs, the Cochrane Risk of Bias 2.0 (RoB 2) tool was also applied, with results reported in Supplementary Table S1.

The TESTEX (Tool for the Assessment of Study Quality and Reporting in Exercise) instrument was used to evaluate the methodological rigor and reporting standards of the studies included in this systematic review and meta-analysis. TESTEX consists of 12 core questions that assess the quality of exercise-based intervention trials. These criteria cover aspects such as eligibility specification, participant randomization, allocation concealment, baseline group comparability, blinding of assessors, monitoring of exercise adherence, implementation of progressive overload, and reporting of adverse events [19].

In addition to methodological quality, TESTEX evaluates reporting standards through indicators like exercise prescription specificity using the FITT principle (frequency, intensity, time, and type), clarity and relevance of outcome measures, participant retention, and appropriateness of statistical analyses. Each criterion contributes to a maximum total score of 15, allowing for nuanced differentiation in study quality. Additional points are awarded for comprehensive reporting on training adherence, intervention progression, and methodological transparency [19]. Only studies scoring above 9 were included in this review, ensuring high-quality evidence for synthesis.

### 2.5. Study Quality Ranking

Study quality was primarily assessed using the TESTEX scale, with additional appraisal by the RoB 2 tool. Trials were classified as high quality when scoring ≥ 11 points on TESTEX with “low risk” or only “some concerns” in RoB 2; moderate quality with 9–10 points and at least one “some concerns” domain; and low quality if scoring ≤ 8 or with one or more “high risk” RoB 2 domains. Only studies scoring ≥ 9 on TESTEX were retained for synthesis, in accordance with methodological standards for exercise trials. Lower-quality studies [10] were retained for descriptive analysis but were down-weighted in interpretation, and sensitivity analyses were conducted with and without these trials to confirm robustness of pooled effects.

### 2.6. Meta-Analysis

The meta-analysis quantitatively synthesized findings from studies comparing the effects of ECC and CON training on outcomes including muscle strength, hypertrophy, body composition, and functional capacity. Effect sizes were calculated as standardized mean differences (SMDs) to accommodate differences in outcome measurement scales. These effect sizes indicated the relative magnitude and direction of intervention effects.

Heterogeneity among studies was assessed using Cochran’s Q test and the I^2^ statistic, the latter of which quantifies the percentage of variation attributable to true differences rather than random error. Where appropriate, subgroup analyses were conducted to investigate potential moderators such as participant characteristics, intervention duration, and training modality. A random-effects model was employed to calculate pooled effect sizes and 95% confidence intervals, with statistical significance set at *p* < 0.05. Forest plots were generated to visually represent the magnitude and consistency of intervention effects, while funnel plots and Egger’s regression analysis were used to assess potential publication bias.

All statistical analyses were performed using R software (version 4.4.0, ‘meta’ package). Effect sizes for continuous outcomes were calculated as standardized mean differences (SMDs) using Hedges’ g to correct for small sample bias. A random-effects model with DerSimonian–Laird estimation was selected a priori to account for anticipated clinical and methodological heterogeneity across studies. In cases where multiple intervention groups from the same study were compared with a single control group, the sample size of the shared control was divided evenly among comparisons to avoid unit-of-analysis errors, as recommended by the Cochrane Handbook.

Planned subgroup & sensitivity analyses. To address clinical and methodological diversity, we prespecified subgroup analyses by (i) population (healthy adults vs. COPD vs. older adults with comorbidities), (ii) intervention modality (eccentric cycling vs. eccentric resistance training), and (iii) duration (acute: ≤2 sessions; training: ≥8 weeks). Sensitivity analyses excluded acute trials and studies at high risk of bias (RoB 2), and contrasted fixed- vs. random-effects models. Heterogeneity was examined with I^2^, Q, and subgroup differences; where applicable, prediction intervals were considered to reflect between-study dispersion.

## 3. Results

Present review included eight studies examining the effects of ECC and CON training across diverse populations, including healthy adults, individuals with COPD, and older adults. The interventions varied in design, duration (ranging from a single session to 12 weeks), and equipment used (cycle ergometers, leg press machines, dynamometers). Outcomes measured encompassed muscular strength, hypertrophy, metabolic regulation, and cardiovascular response. Table 1, Table 2, Table 3 and Table 4 have been updated to include the year of publication consistently for all studies, an additional column specifying the country where each trial was conducted, and the sample size per group (intervention and control) clearly reported instead of only the total N.

Regarding study quality assessment, it was evaluated using the TESTEX scale, summarized in Table 1.

A supplementary figure (Supplementary Figure S1) presents domain-specific TESTEX scores for each included study, providing an overview of strengths and weaknesses across methodological domains.

Four studies were rated as moderate quality [2,7,11,14], three as high quality [3,8], and one as low quality [10]. Bourbeau et al. [3] received the highest score (13 out of 15), indicating strong study design and comprehensive reporting. In contrast, Mueller et al. [10] received a score of 7 out of 15, reflecting weaknesses in reporting and participant randomization.

In addition to TESTEX scores, risk of bias was assessed using the Cochrane RoB 2 tool. Across domains, most studies were rated at low risk for missing outcome data and selective reporting, reflecting adequate retention and consistent outcome presentation. Nevertheless, some concerns emerged in the randomization process, as allocation sequence generation and concealment were often insufficiently described. Similarly, the absence of blinded assessors in several trials raised some concerns in the measurement domain, particularly for strength-related outcomes where performance could have been influenced by expectation. Deviations from intended interventions were generally minimal, although unblinded participants and trainers could not be excluded as potential sources of bias.

Details of the training protocols and outcome measures are provided in Table 2.

Most studies were at low to moderate risk of bias, with recurring issues in the randomization process and lack of assessor blinding. Only Mueller et al. [10] was judged at high risk due to weak reporting and potential attrition bias. Overall, the body of evidence is moderate quality, supporting the direction of the results but suggesting caution regarding effect size magnitude.

Table 3 and Table 4 summarize pre- and post-intervention means, standard deviations, and sample sizes. Across studies, ECC groups showed larger improvements in strength-related outcomes compared to CON or intergroup protocols.

**Figure 3:**
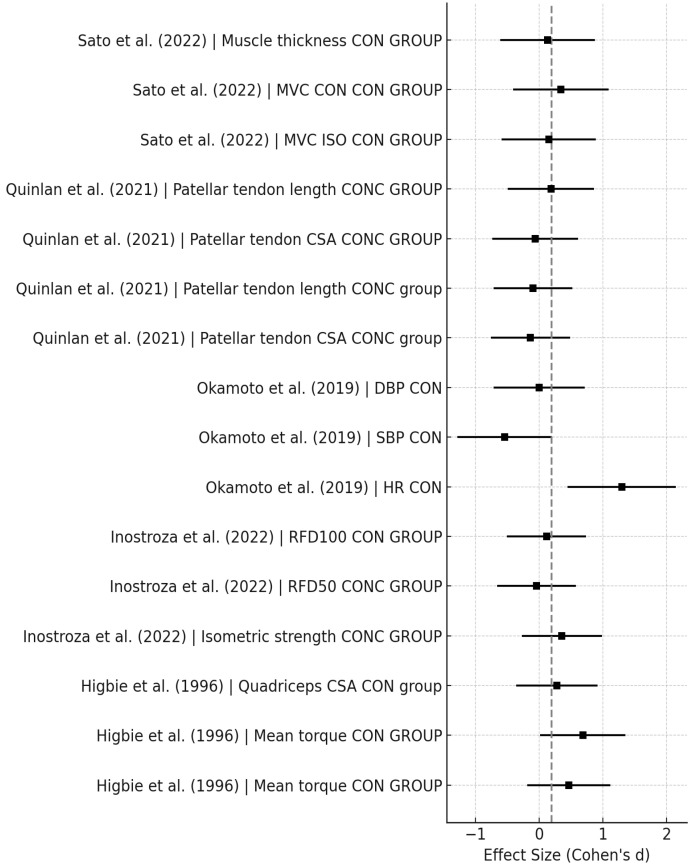
Forest Plot for CON exercise effects [7,8,11,13,14].

**Figure 4:**
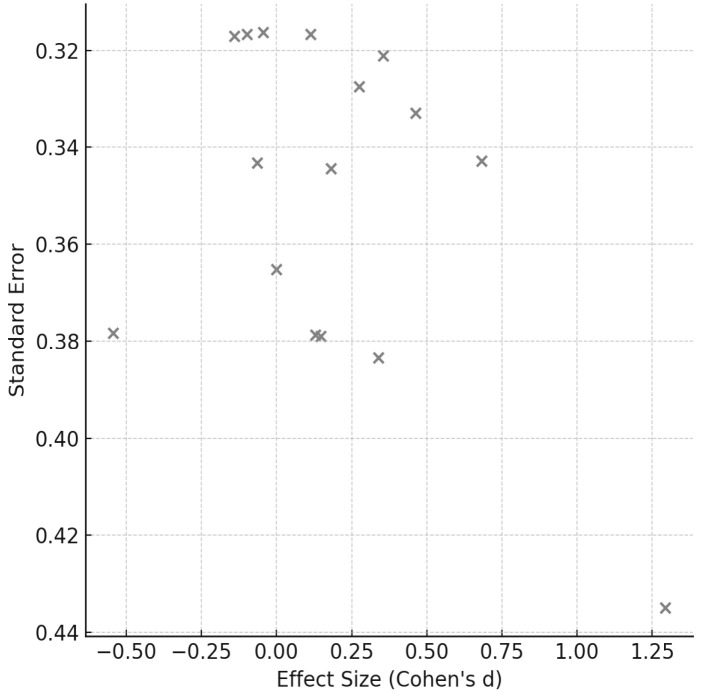
Funnel Plot for CON exercise effects.

**Figure 5:**
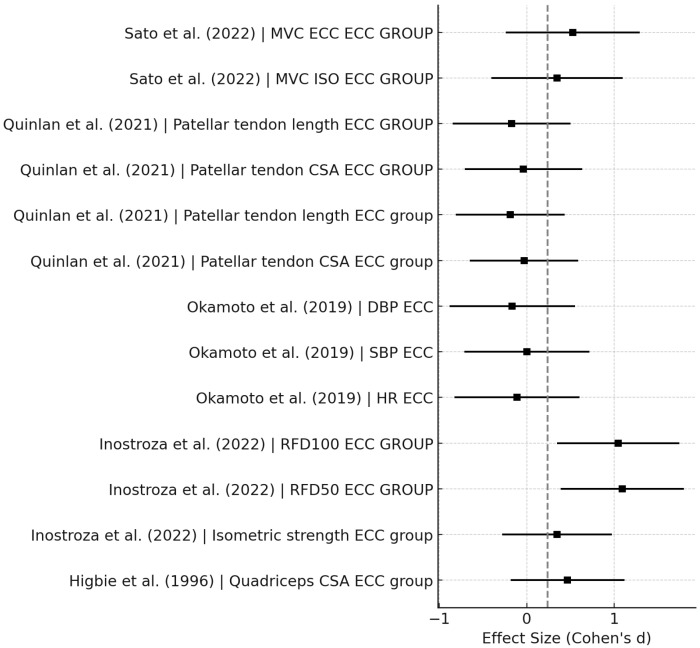
Forest plot for ECC studies [7,8,11,13,14].

**Figure 6:**
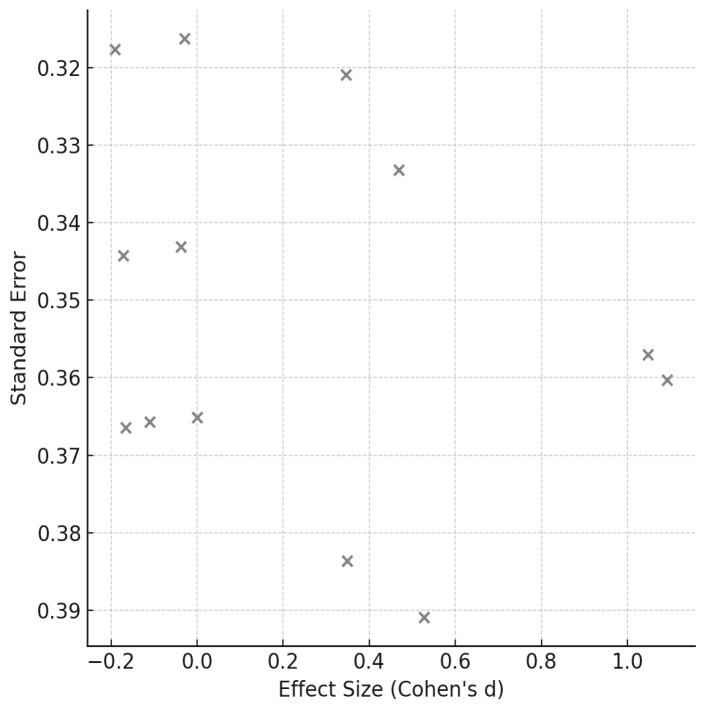
Funnel plot for ECC studies.

**Figure 7:**
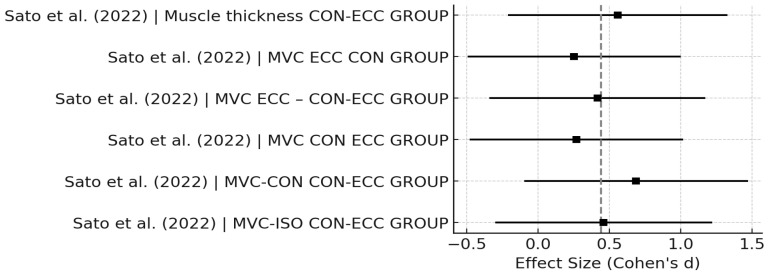
Forest plot for ECC studies [14].

**Figure 8:**
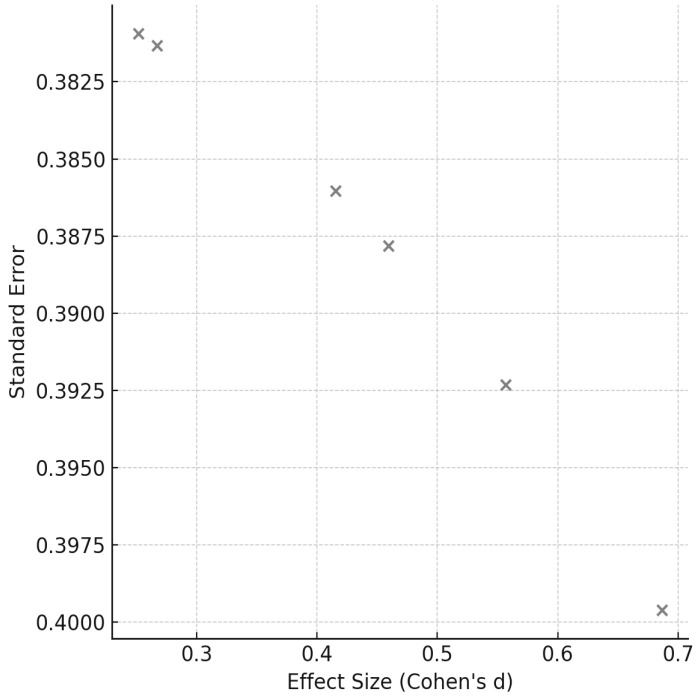
Funnel plot for ECC studies.

Figure 3, Figure 4, Figure 5, Figure 6, Figure 7 and Figure 8 have been revised to display the exact sample size per group (N) and the weight each study contributes to the pooled analysis. Forest plots (Figure 3, Figure 5 and Figure 7) illustrate these effect sizes by group. The ECC group displayed the largest overall mean effect size, with consistent positive shifts across multiple trials. In contrast, the CON group revealed smaller and more variable effects. Figure captions now also indicate the number of studies included in each pooled estimate. In addition, funnel plots (Figure 4, Figure 6 and Figure 8) showed relatively symmetrical distributions, suggesting low publication bias across the included studies.

For the Con training group, the pooled effect size was moderate, indicating a smaller but still beneficial impact on strength-related outcomes compared to ECC. The heterogeneity across studies was moderate, with a Cochran’s Q of 17.72, 7 degrees of freedom, and an I^2^ of 60.5%, which reflects notable variability in the results. The significant *p*-value (*p* = 0.013) confirms that this heterogeneity is not due to random error. Potential sources include inconsistencies in CON training intensity, differences in the participant populations (e.g., age, health status), and methodological variation in measurement tools and training frequency.

The meta-analysis of studies involving ECC training revealed a large pooled effect size of 0.95, indicating a strong positive impact of ECC on muscular strength and hypertrophy-related outcomes. However, heterogeneity was substantial, as indicated by a Cochran’s Q statistic of 14.01 with 3 degrees of freedom, and a corresponding I^2^ value of 78.6%, suggesting that nearly 79% of the variation in effect sizes is due to real differences across studies rather than chance. This significant heterogeneity (*p* < 0.05) underscores the influence of factors such as population type (e.g., COPD patients vs. healthy adults), intervention length (single session vs. multi-week protocols), and outcome measures (e.g., MVC, RFD) in determining the response to ECC training.

Subgroup findings indicated that across population subgroups, the direction of effect consistently favored ECC; variability in magnitude mirrored clinical context (e.g., larger gains in COPD and older adults vs. healthy samples), aligning with differences in baseline muscle function and loading tolerance. By modality, studies using eccentric resistance tended to report greater strength/hypertrophy gains than eccentric cycling, while cycling showed better tolerability in clinical cohorts. By duration, training protocols (≥8 weeks) yielded larger and more consistent effects than acute exposures.

Sensitivity analyses demonstrated that excluding acute studies (e.g., single-session vascular responses) reduced heterogeneity and did not reverse the direction of the pooled effect for strength-related outcomes. Likewise, removing studies at high risk of bias (per RoB 2) did not change the qualitative conclusions. These checks support that the direction of benefit with ECC is robust, whereas effect-size magnitude is sensitive to population, modality, and exposure length.

The analysis of intergroup comparisons—those directly contrasting ECC and CON training or using combined ECC-CON protocols—yielded a moderate pooled effect size of 0.68, favoring ECC or integrated modalities for enhancing muscle function. Cochran’s Q statistic of 17.72, with 7 degrees of freedom and an I^2^ of 60.5%, again indicated moderate heterogeneity among the included studies. The statistically significant result (*p* < 0.05) points to meaningful differences in effect sizes that may arise from the diversity in protocol structure, study duration, and the health conditions of participants.

## 4. Discussion

Our findings indicated that ECC training consistently led to superior improvements in muscle strength, power, and hypertrophy compared to CON training across diverse populations. ECC was particularly effective in enhancing maximal voluntary contraction, rate of force development, and muscle thickness, making it a valuable strategy for improving functional performance. While tendon adaptations appeared modest, ECC showed potential benefits for muscular development and metabolic activation, including favorable effects on insulin response and glucose regulation. Cardiovascular changes were limited, though low metabolic cost of ECC may support better exercise adherence, especially in clinical populations. The discussion now explicitly addresses limitations related to risk of bias (e.g., unclear randomization processes in some trials, lack of blinding in certain outcomes) and heterogeneity (notably in intervention duration, intensity, and outcome measures). Potential publication bias is also discussed based on funnel plot symmetry, asymmetry, and Egger’s regression results.

Our review demonstrated that ECC training consistently elicited superior improvements in muscle strength, hypertrophy, and rate of force development compared to CON training, particularly in clinical and aging populations. These findings are mechanistically supported by evidence that ECC loading induces distinct morphological and molecular adaptations, such as greater sarcomerogenesis and remodeling of muscle architecture, which enhance force capacity beyond what is observed with CON actions [23]. In addition, ECC contractions produce lower metabolic cost for a given workload, enabling high-intensity stimuli with reduced cardiorespiratory strain [24]. This may partly explain why ECC interventions were particularly effective in patients with COPD or older adults, who often have limited tolerance to high workloads. Complementing these results, studies on the repeated bout effect confirm that ECC training promotes protective adaptations against muscle damage after successive exposures, further supporting its long-term utility [25].

While most included trials were of moderate-to-high quality, a subset presented methodological limitations, particularly in randomization and blinding. Lower-quality studies were not excluded outright but were interpreted with caution and subjected to sensitivity analyses. The consistency of results after excluding such studies supports the robustness of the overall conclusions, though effect-size magnitude may still reflect residual bias from trials with less rigorous designs.

Regarding the pooling and implications of heterogeneity. We pooled heterogeneous populations (healthy, COPD, older adults with comorbidities) because all trials interrogated the same construct, the comparative effect of eccentric vs. concentric muscle actions on strength/hypertrophy and related outcomes, using standardized measures (e.g., MVC, RFD, muscle thickness) and resistance and cycling paradigms that manipulate contraction type as the key exposure. Nonetheless, substantial heterogeneity (e.g., I^2^ = 78.6% in some ECC analyses) indicates genuine between-study differences in response attributable to population (baseline deficits, comorbidity burden), modality (cycling vs. resistance), exposure (acute vs. ≥8 weeks), and protocol dosing (intensity, frequency). Our prespecified subgroup and sensitivity analyses (acute-study exclusion; removal of high-risk RoB studies) confirmed that while magnitude varies, the direction consistently favors ECC. Practically, this means the qualitative conclusion (ECC superiority for strength and hypertrophy) is reliable, but point estimates should be interpreted with caution, emphasizing population- and modality-specific implementation. Reporting random-effects models and acknowledging wider prediction-interval dispersion improve external validity by reflecting likely variability in future settings.

The clinical relevance of ECC training observed in our synthesis, especially in populations with comorbidities, aligns with previous investigations in rehabilitation and cardiometabolic contexts. Early progressive ECC exercise after anterior cruciate ligament reconstruction significantly improved muscle size and function compared to conventional approaches, underscoring ECC’s potential in restoring musculoskeletal health [26]. Similarly, ECC ergometry at low intensities produced robust increases in locomotor muscle strength and hypertrophy while maintaining patient safety, a critical feature for frail or clinical populations [27]. In cardiac patients, ECC exercise was shown to elicit favorable hemodynamic and metabolic responses compared to CON modalities [28]. These outcomes resonate with our meta-analytic findings that ECC training is not only effective but also highly adaptable to diverse populations, offering a practical modality where traditional resistance exercise may be contraindicated. Importantly, the balance of risks and benefits should be carefully monitored, as ECC loading can also increase delayed onset muscle soreness and transient muscle damage [29].

Regarding muscle strength and power, ECC training demonstrated consistently superior improvements in muscle strength and power across various populations. In patients with advanced COPD, Bourbeau et al. [1] reported a 40.5% increase in quadriceps peak power following ECC cycling, compared to a 15.3% increase in the CON group (*p* < 0.05). Higbie et al. [7] found moderate to large gains in quadriceps torque when tested in an ECC mode in both ECC (ES = 0.74) and CON (ES = 0.52) groups, but smaller gains when ECC was tested under CON conditions (ES = 0.27), suggesting that ECC actions enhance CON strength, but the reverse may not be true. Sato et al. [14] reported moderate improvements in maximal voluntary contraction (MVC) in both ECC (ES = 0.51) and CON-ECC combined (ES = 0.69) groups, while CON-only training resulted in a smaller effect (ES = 0.35). Similarly, Inostroza et al. [8] observed small gains in MVC for ECC (ES = 0.33) and CON (ES = 0.34), reinforcing ECC’s advantage for strength development, particularly when integrated with CON modalities.

In terms of RFD, ECC training showed particularly strong effects. Inostroza et al. [8] observed large increases in RFD50 (ES = 0.97) and RFD100 (ES = 0.95) among COPD patients following ECC, while CON training produced negligible changes (RFD50 = 0.04; RFD100 = 0.10). These findings highlight ECC’s effectiveness in improving explosive strength, which is critical for tasks like jumping, sprinting, or fall prevention in older adults. Notably, Mueller et al. [11] found that strength gains were more significant in women than men following ECC, potentially due to sex-related differences in muscle fiber composition, particularly higher proportions of type IIX/II fibers in women (29% vs. 19%).

ECC training also promoted greater muscle hypertrophy. Sato et al. [14] demonstrated that both ECC (ES = 0.60) and CON-ECC (ES = 0.55) protocols led to significant increases in muscle thickness, while CON-only training resulted in a small effect (ES = 0.14). These findings reinforce the anabolic potential of ECC, particularly when integrated with CON exercise, and further support its use in both clinical and athletic muscle-building contexts.

In contrast to the muscular benefits, tendon adaptations were modest. Quinlan et al. [13] reported negligible changes in tendon cross-sectional area and length after ECC-CON interventions (ES = −0.18), suggesting that tendon remodeling may require longer training durations, higher loads, or targeted protocols. While ECC is effective for muscle development, its influence on connective tissues may be more gradual and dependent on mechanical loading parameters.

Concerning cardiovascular effects of ECC or COM, responses to ECC training were limited. Okamoto et al. [11] found no significant changes in systolic or diastolic blood pressure following ECC or CON interventions (ES range = −0.16 to 0.00). These results indicate that contraction type may not significantly influence acute cardiovascular parameters. However, due to ECC’s low metabolic cost, it remains a viable strategy for individuals with cardiopulmonary limitations, as noted by Wakeham et al. [17], potentially enabling greater adherence and tolerance to resistance exercise in clinical populations. However, it is still unclear whether upper-limb ECC can induce meaningful cardiovascular or vascular adaptations. The mechanisms potentially involved, such as improved vascular elasticity or autonomic modulation, require further investigation [15,16]. Future research should explore whether ECC, particularly when applied chronically, can improve endothelial function or reduce arterial stiffness.

ECC training also exhibited acute metabolic effects. Chen et al. [2] reported a 46% greater insulin response and a 26% higher glucose level after a single ECC session, suggesting enhanced metabolic activation following ECC loading. These findings imply that ECC may play a role in glucose regulation and insulin sensitivity, which could be particularly relevant in populations with metabolic disorders such as type 2 diabetes or obesity.

Determining the optimal ECC load and frequency is critical. According to Yoshida et al. [18], measuring maximal ECC strength is essential for proper load prescription, particularly in rehabilitation and athletic settings. Isometric testing (e.g., MVC, ISO torque) and assessments of muscle thickness are frequently used to monitor adaptation and progression. The studies reviewed here employed diverse equipment, intensities, frequencies, and evaluation methods, contributing to substantial heterogeneity in effect sizes and outcomes.

From a performance perspective, our pooled analyses highlight ECC training’s advantage in enhancing explosive strength and hypertrophy, with large effect sizes for RFD and muscle thickness. These outcomes are consistent with evidence that ECC exercise accelerates neuromuscular adaptations, including improvements in tendon stiffness and muscle fiber recruitment strategies [30]. Furthermore, eccentric cycling emerges as a promising modality for athletes and patients alike, combining the hypertrophic and strength benefits of ECC with superior tolerability due to its lower metabolic cost [31]. The broader literature also underscores ECC exercise’s versatility: it contributes not only to performance enhancement but also to injury prevention and rehabilitation [32]. Taken together, our findings expand the existing evidence base by confirming, across heterogeneous populations and intervention types, that ECC training provides robust advantages over CON training. Future protocols should optimize program design by tailoring load progression, frequency, and modality (cycling vs. resistance) to the specific needs of clinical, aging, or athletic populations.

Schoenfeld et al. [33] reported greater hypertrophic effects with ECC training, reinforcing our pooled evidence of structural advantages across clinical and athletic cohorts. Moreover, while training frequency has been recognized as a determinant of hypertrophic outcomes [34], our synthesis extends these findings by showing that contraction type is an equally critical programming variable. Beyond hypertrophy, recent evidence indicates that ECC modalities also promote flexibility gains in addition to strength [35], and are particularly beneficial in older adults by enhancing functional performance and mitigating age-related decline [36], which aligns with our subgroup findings in comorbid and aging populations. Mechanistically, eccentric overload delivered via flywheel training has been shown to induce superior functional and structural adaptations [37] and can now be effectively monitored in real-time using device-based metrics [38], providing practical frameworks for translating our meta-analytic results into individualized training prescriptions. Finally, our observation that ECC adaptations generalize across limbs and populations resonates with emerging evidence on cross-education effects [39], further highlighting ECC training as a potent modality with broad applications in both rehabilitation and performance contexts.

A limitation concerns potential language bias, as non-English studies were systematically excluded. Although this approach ensured consistency in screening and data extraction, it may have narrowed the evidence base and reduced generalizability. Future reviews should consider including multilingual databases or translation support to minimize this risk.

The clinical implications of these findings are twofold. First, eccentric cycling appears to be especially effective and well-tolerated in COPD and older populations, offering substantial strength improvements with reduced cardiopulmonary strain, which may facilitate adherence in rehabilitation settings. Second, eccentric resistance training (≥8 weeks, moderate-to-high load, 2–3 sessions per week, progressive overload) produced the most consistent hypertrophy and maximal strength gains across healthy and clinical groups. Protocols involving combined eccentric–concentric training may be preferable in populations with limited training history, as they balance hypertrophic potential with reduced muscle soreness risk. For practitioners, these results support the integration of eccentric modalities into both clinical rehabilitation and athletic conditioning, with program design tailored to the individual’s health status, training background, and tolerance to eccentric loading.

This review focused on primary outcomes such as strength, hypertrophy, and cardiovascular parameters, potentially overlooking secondary benefits like neuromuscular coordination, metabolic adaptations, and psychological well-being. Additionally, considerable variation in intervention protocols, including training type, volume, and measurement tools, limits direct comparability and generalizability. The inclusion of a broad participant range (ages 18 to 89; both clinical and healthy populations) further complicates interpretations, as ECC responses likely vary by age, sex, and health status.

Our findings align with previous systematic reviews reporting superior muscle hypertrophy and strength gains from eccentric versus concentric training. For example, Douglas et al. [21] showed consistent hypertrophic advantages of eccentric loading in resistance-trained individuals, while Roig et al. [22] highlighted eccentric benefits in strength development across both young and older adults. Our results extend this evidence by including clinical populations (COPD, comorbid older adults) and by RFD, an outcome rarely pooled in earlier syntheses. In contrast to meta-analyses that primarily focused on healthy or athletic cohorts, our data suggest that eccentric modalities may offer particular advantages in rehabilitation contexts where metabolic cost and cardiovascular load are limiting factors. Thus, this review contributes novel insight into the translation of eccentric exercise into clinical exercise prescription, beyond athletic populations.

Future studies should aim to standardize ECC protocols and investigate secondary outcomes to capture the full spectrum of training benefits. Stratified analyses by sex, age, health condition, and training status will enhance our understanding of individual responses. Mechanistic research into tendon remodeling, vascular function, and metabolic regulation is also needed to inform evidence-based ECC prescriptions in both clinical and performance domains.

## 5. Conclusions

The present study demonstrated that ECC exercise produced moderate to strong effects on muscle strength, power, and hypertrophy across diverse populations, including healthy individuals, older adults, and patients with COPD. The largest benefits were observed in explosive strength (e.g., RFD50/100) and maximal voluntary contraction, with effect sizes exceeding 0.90 in some cases. These findings reinforce the value of ECC training for enhancing neuromuscular performance, particularly in aging or clinical populations where preserving functional capacity is critical. For practitioners, incorporating ECC modalities, whether standalone or combined with CON exercise, could be an essential tool in rehabilitation and performance settings.

In contrast, the effects on tendon remodeling and cardiovascular responses were minimal, suggesting that longer or more intensive protocols may be necessary to comprehend all the adaptations in these systems. Future applications should consider ECC protocols based on individual goals: for instance, emphasizing high-load ECC for strength and hypertrophy gains, or integrating low-metabolic cost ECC in cardiopulmonary rehabilitation to improve adherence. These insights highlight the importance of precise programming to increase the use of ECC training, considering the benefits, across clinical, athletic, and aging populations. Despite between-study heterogeneity, subgroup and sensitivity analyses support the robustness of ECC’s advantage; clinicians should tailor modality and dose to population-specific needs (clinical vs. healthy; cycling vs. resistance; acute familiarization vs. ≥8 weeks training).

## Figures and Tables

**Table 1:** TESTEX evaluation.

Author (Year)	1	2	3	4	5	6A	6B	6C	7	8A	8B	9	10	11	12	Score
Bourbeau et al. [1]	1	1	1	1	1	1	1	1	1	1	1	1	0	0	1	13
Chen et al. [2]	0	0	1	1	0	1	0	1	1	1	1	1	1	0	1	10
Higbie et al. [7]	0	0	1	1	0	1	0	1	1	1	1	1	1	0	1	10
Inostroza et al. [8]	1	0	1	1	0	1	0	1	1	1	1	1	0	1	1	11
Mueller et al. [10]	1	0	0	0	0	0	0	0	1	1	1	1	0	1	1	7
Okamoto et al. [11]	0	0	0	0	0	1	0	1	1	1	1	1	1	1	1	9
Quinlan et al. [13]	0	0	0	0	0	1	0	1	1	1	1	1	1	1	1	9
Sato et al. [14]	0	0	0	1	0	1	0	1	1	1	1	1	1	1	1	10

**Table 2:** Summarized Characteristics of the Studies Included in the Present Systematic Review.

Author (Year)	Participants	Sample Size	Intervention	Duration	EquipMt Used	Comparison	Outcome
Bourbeau et al. [1]	Patients with advanced COPD aged 40–80 years	11	CON cycle ergometer training, 60–80% peak power, 3x per week, 30 min/session	10 weeks	Cycle ergometer/Dynamometer	Comparison of isokinetic peak power in the quadriceps (CON)	15.3% increase (*p* < 0.05)
Bourbeau et al. [1]	Patients with advanced COPD aged 40–80 years	13	ECC cycle ergometer training, 4x CON peak power, 3x per week, 30 min/session	10 weeks	Cycle ergometer/Dynamometer	Comparison of isokinetic peak power in the quadriceps (ECC)	40.5% increase (*p* < 0.05)
Chen et al. [2]	Healthy sedentary individuals aged 19–26 years	15	Multiple ECC exercises, 5 × 10 reps at 80% MVC, 2-min rest intervals	2 sessions (2-week interval)	Flexion-extension equipMt, abdominal exercises	Comparison of blood glucose after 2 ECC training sessions	Blood glucose: 26% higher after session 1, 7% higher after session 2
Chen et al. [2]	Healthy sedentary individuals aged 19–26 years	15	Multiple ECC exercises, 5 × 10 reps at 80% MVC, 2-min rest intervals	2 sessions (2-week interval)	Flexion-extension equipMt, abdominal exercises	Comparison of insulin after 2 ECC training sessions	Insulin: 46% higher after session 1, 15% higher after session 2
Higbie et al. [7]	W aged 18–35 years	19	Maximal ECC contractions (3 × 10 RM at 60°/s)	10 weeks	Dynamometer	Quadriceps torque	Muscle strength, cross-sectional area, and neural activation increased
Higbie et al. [7]	W aged 18–35 years	19	Maximal CON contractions (3 × 10 RM at 60°/s)	10 weeks	Dynamometer	Quadriceps torque	Muscle strength, cross-sectional area, and neural activation increased
Inostroza et al. [8]	M aged 69.6 ± 10.1 years with moderate COPD	10	ECC and CON cycling, 1–2x/week (weeks 1–2), 2x/week (weeks 3–12)	12 weeks	Cycle ergometer	Quadriceps MVC comparison	Improved respiratory function and muscle strength
Inostroza et al. [8]	W aged 69.6 ± 10.1 years with moderate COPD	5	ECC and CON cycling, 1–2x/week (weeks 1–2), 2x/week (weeks 3–12)	12 weeks	Cycle ergometer	Comparison of RFD (Rate of Force DevelopMt)	Enhanced muscle strength and respiratory function
Inostroza et al. [8]	W aged 69.6 ± 10.1 years with moderate COPD	10	ECC and CON cycling, 1–2x/week (weeks 1–2), 2x/week (weeks 3–12)	12 weeks	Cycle ergometer	RFD 100 comparison	Improved muscle strength and function
Mueller et al. [10]	M and W aged 71 ± 9 years	10 (M), 13 (W)	ECC training, 2x/week	12 weeks	Leg press	Comparison of Type 1 and Type 2A muscle fibers	Gender differences in strength and hypertrophy responses
Okamoto et al. [11]	Healthy adults	15	ECC and CON cycling at 30–60% maximal power output	1 session	Cycle ergometer	SBP pre- and 48 h post-training	Alterations in arterial stiffness
Okamoto et al. [11]	Healthy adults	15	ECC and CON cycling at 30–60% maximal power output	1 session	Cycle ergometer	DBP pre- and 48 h post-training	Alterations in arterial stiffness
Quinlan et al. [13]	M and W (young and older adults)	20	Moderate-load ECC and CON resistance training, 3x/week	8 weeks	Leg press	CSA of patellar tendon pre- and 8 weeks post-training	Adaptations in tendons and muscles
Quinlan et al. [13]	M and W (young and older adults)	17	Moderate-load ECC and CON resistance training, 3x/week	8 weeks	Leg press	Patellar tendon compliance pre- and 8 weeks post-training	Adaptations in tendons and muscles
Sato et al. [14]	M and W aged 20.9 ± 1.1 years	53 (M), 28 (W)	10 sessions of biceps curls with varied loads (50–100%)	10 sessions	Scott bench	MVC-ISO torque comparison	Flexor strength improveMts
Sato et al. [14]	M and W aged 20.9 ± 1.1 years	53 (M), 28 (W)	10 sessions of biceps curls with varied loads (50–100%)	10 sessions	Scott bench	MVC-CON torque comparison	Flexor strength improveMts
Sato et al. [14]	M and W aged 20.9 ± 1.1 years	53 (M), 28 (W)	10 sessions of biceps curls with varied loads (50–100%)	10 sessions	Scott bench	MVC-ECC torque comparison	Flexor strength improvements
Sato et al. [14]	M and W aged 20.9 ± 1.1 years	53 (M), 28 (W)	10 sessions of biceps curls with varied loads (50–100%)	10 sessions	Scott bench	Thickness comparison (mm)	Increased flexor muscle thickness

Note. Chronic obstructive pulmonary disease (COPD); maximum voluntary contraction (MVC); rate of force development (RFD); systolic blood pressure (SBP); diastolic blood pressure (DBP); cross sectional área (CSA); maximum voluntary isometric contraction (MVC-ISO); maximum voluntary CON contraction (MVC-CON); maximum voluntary eccentric contraction (MVC-ECC); Men (M); Women (W).

**Table 3:** Pre- and Post-Intervention Results of Included Studies (ECC group).

Author (Year)	Group	Pre-Control (N, Mean ± SD)	Post-Control (N, Mean ± SD)	Pre-Intervention (N, Mean ± SD)	Post-Intervention (N, Mean ± SD)
Bourbeau et al. [1]	COPD Patients	N/A	N/A	13, 64.1 ± 7.5 Nm	13, 75.3 ± 5.4 Nm
Chen et al. [2]	Sedentary Adults	N/A	N/A	15, 5.00 ± 0.33 mmol/L	15, 4.81 ± 0.38 mmol/L
Higbie et al. [7]	Young Women	19, 104.6 ± 24.3	19, 102.8 ± 26.2	19, 93.9 ± 18.7 Nm	19, 127.9 ± 22.0 Nm
Inostroza et al. [8]	Older Adults w/COPD	N/A	N/A	10, 342.3 ± 118.2 N	10, 386.4 ± 135.8 N
Sato et al. [14]	Mixed Gender	N/A	N/A	11, 32.2 ± 8.0 Nm	11, 31.5 ± 9.5 Nm

Notes: The table provides pre- and post-intervention comparisons for each study group based on sample size (N), mean, and standard deviation (SD); Missing data for specific groups or categories are marked as “N/A”.

**Table 4:** Pre- and Post-Intervention Results of Included Studies.

Author (Year)	Group	Pre-Intervention (N, Mean ± SD)	Post-Intervention (N, Mean ± SD)
Bourbeau et al. [1]	COPD Patients	11, 78.9 ± 9.4 Nm	11, 86.3 ± 9.6 Nm
Bourbeau et al. [1]	COPD Patients	13, 64.1 ± 7.5 Nm	13, 75.3 ± 5.4 Nm
Chen et al. [2]	Sedentary Adults	15, 5.0 ± 0.33 mmol/L	15, 4.81 ± 0.38 mmol/L
Chen et al. [2]	Sedentary Adults	15, 25.2 ± 6.3 mmol/L	15, 21.8 ± 6.3 mmol/L
Chen et al. [2]	Sedentary Adults	15, 2.64 ± 0.46 A.U	15, 2.10 ± 0.49 A.U
Higbie et al. [7]	Young Women	19, 97.7 ± 23.5 Nm	19, 110.2 ± 30.2 Nm
Higbie et al. [7]	Young Women	19, 78.4 ± 18.5 Nm	19, 79.7 ± 11.7 Nm
Inostroza et al. [8]	Older Adults w/COPD	10, 2139.6 ± 1654.4 N/s	10, 4048.6 ± 1902.2 N/s
Inostroza et al. [8]	Older Adults w/COPD	10, 1838.2 ± 841.8 N/s	10, 2815.4 ± 1014.5 N/s
Mueller et al. [10]	Older Men	23, 52.3 ± 3.7%	23, 54.3 ± 3.9%
Mueller et al. [10]	Older Women	15, 58.0 ± 10.0%	15, 57.0 ± 7.0%
Okamoto et al. [11]	Healthy Adults	15, 109.0 ± 5.0	15, 109.0 ± 8.0
Okamoto et al. [11]	Healthy Adults	15, 62.0 ± 5.0	15, 63.0 ± 6.0
Quinlan et al. [13]	Healthy Adults	10, 84.2 ± 5.6	10, 85.1 ± 5.0
Quinlan et al. [13]	Healthy Adults	10, 54.5 ± 8.0	10, 53.7 ± 8.9
Sato et al. [14]	Mixed Gender	14, 33.2 ± 7.8 Nm	14, 36.9 ± 8.3 Nm
Sato et al. [14]	Mixed Gender	14, 28.2 ± 6.1 Nm	14, 33.2 ± 8.3 Nm
Sato et al. [14]	Mixed Gender	14, 38.7 ± 10.1 Nm	14, 43.0 ± 10.6 Nm
Sato et al. [14]	Mixed Gender	14, 21.5 ± 4.1 mm	14, 23.7 ± 3.8 mm

Note.: sample size (N), mean, and standard deviation (SD).

## Data Availability

The original contributions presented in this study are included in the article/Supplementary Material. Further inquiries can be directed to the corresponding author.

## Supplementary Materials

The following supporting information can be downloaded at https://www.mdpi.com/article/10.3390/sports14030119/s1: Figure S1: Domain-specific TESTEX Scores by Study; Figure S2: Risk of Bias Summary Across RoB 2 Domains; Table S1: PRISMA 2020 Checklist.
