# Supplementary material for: Eccentric vs. Concentric Training: A Systematic Review and Meta-Analysis of Randomized Controlled Trials on Performance and Health Benefits Across Diverse Populations
Source: Sports (Basel). 2026 Mar 18;14(3):119. doi: 10.3390/sports14030119 (PMC13030668; doi:10.3390/sports14030119)
Supplement: Supplementary file 1 [file sports-14-00119-s001.zip › supplementary carol.pdf]

**Data Availability Statement:**

**Conflicts of Interest:** The authors declare no conflicts of interest.

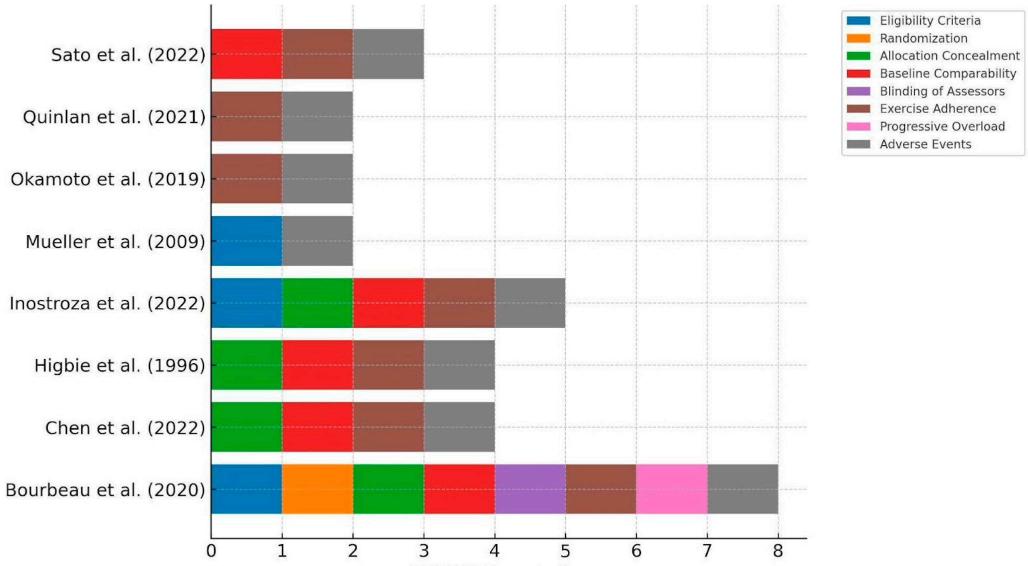


**Figure S1.** Domain-specific TESTEX Scores by Study.

| Study | Randomization | Deviations | Missing data | Measurement | Selection | Overall |
|---|---|---|---|---|---|---|
| Bourbeau et al. (2020) | Some concerns | Low risk | Low risk | Low risk | Low risk | Low risk – overall reliable |
| Chen et al. (2017) | Some concerns | Some concerns | Low risk | Some concerns | Low risk | Some concerns |
| Higbie et al. (1996) | Some concerns | Some concerns | Low risk | Some concerns | Low risk | Some concerns |
| Inostroza et al. (2022) | Low risk | Low risk | Low risk | Some concerns | Low risk | Low to moderate risk |
| Mueller et al. (2009) | High risk | Some concerns | Some concerns | Some concerns | Some concerns | High risk |
| Okamoto et al. (2019) | Some concerns | Some concerns | Low risk | Some concerns | Some concerns | Some concerns |
| Quinlan et al. (2021) | Low risk | Low risk | Low risk | Low risk | Some concerns | Low risk – overall reliable |
| Sato et al. (2022) | Low risk | Low risk | Low risk | Some concerns | Low risk | Low risk |

**Figure S2.** Risk of Bias Summary Across RoB 2 Domains.
